# A new band selection approach integrated with physical reflectance autoencoders and albedo recovery for hyperspectral image classification

**DOI:** 10.1038/s41598-025-09355-7

**Published:** 2025-07-31

**Authors:** V. Sangeetha, L. Agilandeeswari

**Affiliations:** School of Computer Science Engineering & Information Systems, Vellore Institute of Technology, Vellore, 632014 India

**Keywords:** Dimensionality reduction, Autoencoders, Electromagnetic spectrum, Hyperspectral classification, Computer science, Environmental impact

## Abstract

Hyperspectral imaging has emerged as a powerful tool for remote sensing applications, offering rich spectral information across a broad electromagnetic spectrum. However, the high dimensionality of hyperspectral data poses significant challenges in analysis and interpretation. In this study, we propose a novel approach for hyperspectral image processing, focusing on dimensionality reduction, albedo recovery, and subsequent classification. Our method begins with a grouping strategy based on the electromagnetic spectrum that considers the images’ physical properties, facilitating the segmentation of hyperspectral data into meaningful spectral bands. This grouping reduces the dimensionality of the data and preserves crucial spectral information. Subsequently, we integrate autoencoders to incorporate non-linear transformations in the feature extraction phase, thereby improving the model’s capacity to learn intricate patterns within the data. A key goal of our methodology is to effectively embed spatial information into the representation. Albedo recovery is employed aimed at improving spatial resolution while retaining spectral fidelity. By leveraging the reduced-dimensional representation obtained through grouping and autoencoders, we reconstruct the hyperspectral image with enhanced spatial details, thereby facilitating more accurate interpretation and analysis. To assess the performance of the proposed approach, we perform experiments using three standard hyperspectral datasets: Indian Pines, University of Pavia, and Salinas.

## Introduction

**Fig. 1:**
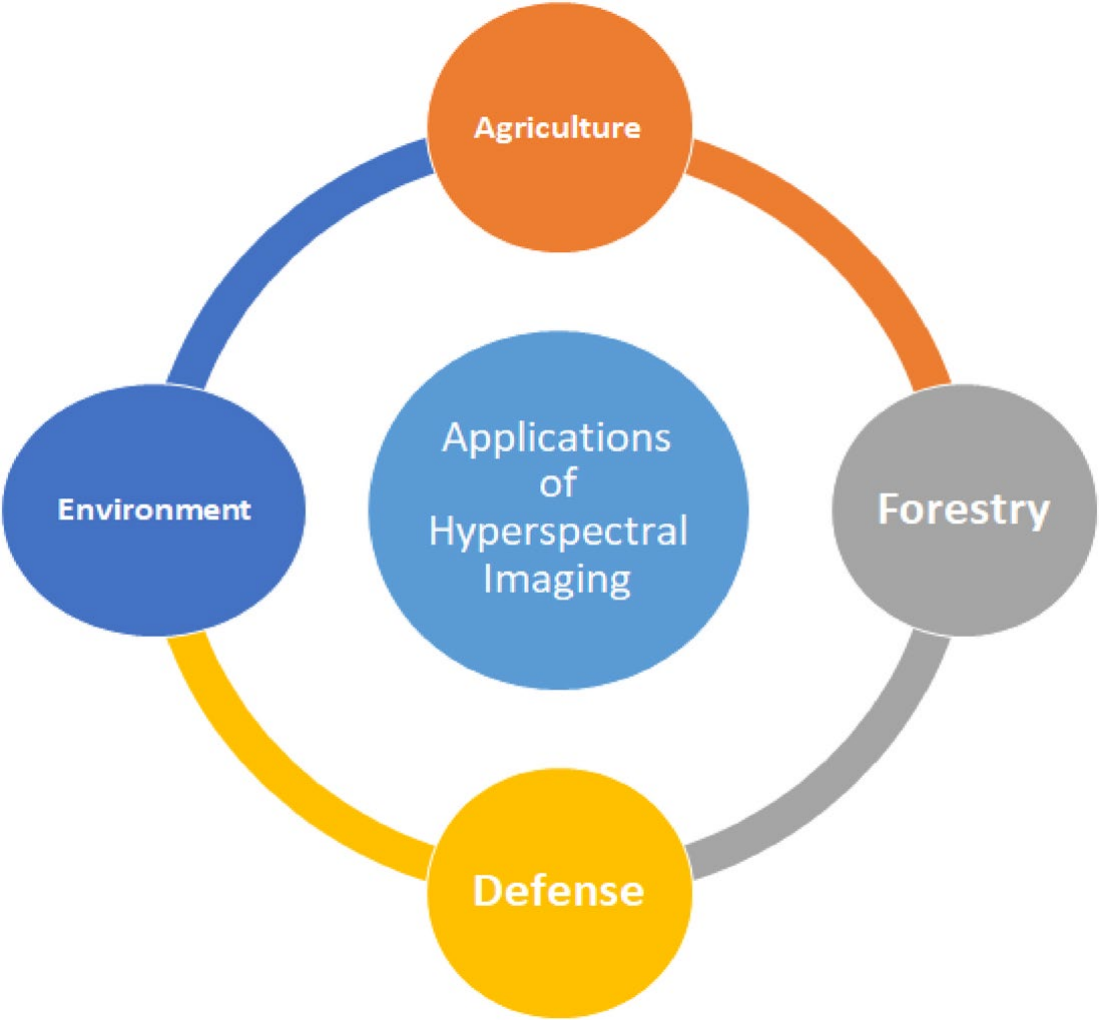
Applications of Hyperspectral Imaging.

One of the most challenging tasks for hyperspectral images is the allotment of labels to pixels and turn out to be classification^1^. The hyperspectral images contain abundant data, which requires processing before the assignment of a label^2^. These images are acquired through hyperspectral sensors. The images are widely used in different real-time applications, as shown in Fig. 1, such as health care, medical treatment, the healthiness of fruits, drug composition, crop identification^3^, military^4^, and mineral exploration^5^. Even though the applications are widespread, these images contain fewer training samples, high correlation, and redundancy. Dimensionality reduction (DR) is a crucial and necessary step for effective hyperspectral image (HSI) data processing, such as classification^6^. There are two primary DR techniques in the literature: feature/band selection (BS) and feature extraction.   Band selection (BS) techniques are generally divided into supervised, semi-supervised, and unsupervised categories^7^. While supervised and semi-supervised methods depend on labeled training data, acquiring such labels for hyperspectral imagery (HSI) is often difficult. As a result, unsupervised BS methods are commonly favored^8^. The primary types of BS methods include clustering-based approaches, ranking strategies, search-based techniques, combinatorial optimization methods, and hybrid frameworks^9^.

Brief discussion of each type of BS approach^10,11^, as explained below:

- *Ranking-based methods* These techniques choose the top-ranked bands by giving each band a score based on factors like mutual information, information entropy, or statistical separability metrics (such as Bhattacharyya distance). They frequently overlook inter-band dependencies despite being computationally efficient.
- *Search-based techniques* These techniques use heuristic or metaheuristic search algorithms (such as ant colony optimization, simulated annealing, or genetic algorithms) to find the best subset of bands that optimizes a classification or clustering goal. Although they can perform competitively, they are typically computationally demanding, particularly when dealing with high-dimensional data.
- *Combinatorial optimization-based approaches* These approaches BS as an optimization problem, which is frequently expressed as submodular maximization, convex optimization, or integer programming. Despite having a theoretical foundation, they may have scalability problems and require complex solvers.
- *Hybrid approaches* These combine two or more of the aforementioned techniques, for example, search-based fine-tuning with ranking-based filtering. The goal of hybrid approaches is to strike a compromise between computational efficiency and performance.
- *Grouping-based techniques (our main area of interest)* These methods choose representative bands from each group and cluster bands according to similarity (e.g., correlation, distance metrics, graph representations). They are especially good at reducing redundancy and maintaining the spectral structure.

Grouping-based methods for band selection (BS) have attracted significant attention in prior research. For example, in^12^, researchers have proposed various advanced strategies for hyperspectral band selection. One such method is the Adaptive Subspace Partition Strategy (ASPS), which segments the hyperspectral image cube into multiple sub-cubes by optimizing the ratio between inter-class and intra-class distances. Building on this, a subsequent approach known as Fast Neighborhood Grouping for Band Selection (FNGBS)^13^ was introduced. FNGBS adopts a coarse-to-fine grouping technique, clustering the hyperspectral cube and selecting bands from each group based on the highest combined value of local density and information entropy. More recently, another method called Optimal Neighborhood Reconstruction (ONR)^14^ was developed, utilizing the Correlated Neighborhood Property (CNP) to exploit strong inter-band correlations, thereby enhancing the selection of informative bands.

Conversely, feature extraction and its related techniques apply transformation functions that alter the inherent structure of the data, enabling the representation to capture more meaningful and discriminative patterns^49–51,53,55–58^. The realm of hyperspectral image classification has seen a surge in interest regarding non-linear feature extraction methods. These techniques are pivotal in extracting intricate patterns and relationships within hyperspectral data, which often exhibit nonlinear characteristics. Unlike their linear counterparts, nonlinear feature extraction methods delve into the complexities of hyperspectral data, leveraging sophisticated mathematical frameworks to uncover hidden structures. Research in this domain has explored a plethora of methodologies, including kernel methods, neural networks, and manifold learning techniques. Kernel-based techniques, including kernel principal component analysis (PCA)^15,28^ and kernel Fisher discriminant analysis (FDA)^16^, are effective in modeling nonlinear patterns in hyperspectral data by projecting the input into a high-dimensional feature space where complex relationships become more discernible. Furthermore, manifold learning techniques such as t-distributed Stochastic Neighbor Embedding (t-SNE)^12,47^ and Isomap aim to retain both local and global geometric structures of the data. These methods enable effective nonlinear feature extraction while reducing the impact of the curse of dimensionality. Literature in this field showcases a rich tapestry of studies, each contributing novel insights and methodologies to advance the efficacy of nonlinear feature extraction for hyperspectral image classification. By harnessing these methods, researchers aim to unlock the full potential of hyperspectral data, leading to enhanced classification accuracy and a deeper understanding of complex spectral signatures in various applications, from environmental monitoring to precision agriculture and beyond.

By combining deep learning architectures with innovative feature extraction methods, hyperspectral image (HSI) classification has advanced significantly in recent years. Notably, PFS3F: Probabilistic Fusion of Superpixel-wise and Semantic-aware Structural Features was presented in the work by^17^. This technique successfully integrates semantic structural features and superpixel segmentation to improve classification accuracy in complicated scenarios. This probabilistic fusion method performs better on many HSI benchmarks by utilizing both spatial and semantic information.

Another encouraging advancement was made by^18^, who combined Transformers with SimAM-based Convolutional Neural Networks (CNNs) to offer a quick and effective HSI classification framework. This hybrid model achieves state-of-the-art outcomes at a lower computing cost by utilizing CNNs’ spatial feature refinement and Transformers’ global contextual modeling capabilities.

A Dual-Branch Structural Feature Extraction Method was created more recently by^19^ to extract both local and global structural features from hyperspectral data. Their approach successfully represents multi-scale spatial-spectral information by utilizing a dual-branch design, which improves classification performance on difficult datasets.

This research demonstrates the expanding trend of using multi-scale feature fusion and hybrid models to overcome the problems with HSI data, like spectrum variability and high dimensionality. By introducing a spectral-partitioned autoencoder-based dimensionality reduction strategy that preserves important spectral information across visible, near-infrared (NIR), and shortwave infrared (SWIR) bands, our proposed method adds to this changing landscape. A convolutional neural network is then used to efficiently classify the reduced spectral-spatial representation.

Numerous dimensionality reduction methods existing in the literature have focused exclusively on either feature extraction or band selection methods. To improve classification performance, the present method exploits the advantages of both nonlinear feature extraction and grouping-based band selection properties.

The key limitations of current approaches are neglect of physical properties^20^, Overemphasis on Either Spatial or Spectral Features^21]– [22^, and lack of robustness across datasets^23^. the discussion on the limitations of existing BS methods provides empirical evidence that supports the need for approaches that consider both the physical properties of spectral bands and the integration of spatial-spectral features. This, in turn, reinforces the motivation for the proposed method.

Furthermore, the key highlights of the present work are stated below.

1. The introduction of a nonlinear dimensionality reduction technique was created especially for HSI classification.
2. A physically guided spectral segmentation technique is implemented using the VISIBLE, NIR, and SWIR bands.
3. Comprehensive empirical validation on benchmark and real-world datasets, demonstrating improved performance over the most cutting-edge methods.

 The remainder of the paper is structured as follows: Sect. 2 outlines the technical aspects of the proposed framework, while Sect. 3 describes the benchmark datasets used in the study. Sect. 4 presents experimental results along with parameter analysis. Sect. 5 provides a detailed discussion and interpretation of the results, and finally, Sect. 6 concludes the paper.

## Proposed framework

This section is dedicated to the Mathematical description of the proposed method. Let the HSI dataset be $\:\text{H}\in\:{\text{R}}^{\text{h}\times\:\text{w}\times\:\text{d}}$ where $\:\text{h}\text{*}\text{w}$ denotes spatial information and d represents the number of spectral bands. According to spectral reflectance properties in various regions of the electromagnetic spectrum, HSI data can be separated into three partitions $\:{H}_{visible}\in\:{\text{R}}^{\text{h}\times\:\text{w}\times\:{l}_{1}}$ in the VISIBLE region, $\:{H}_{NIR}\in\:{\text{R}}^{\text{h}\times\:\text{w}\times\:{l}_{2}}$ in the NIR region and $\:{H}_{swir}\in\:{\text{R}}^{\text{h}\times\:\text{w}\times\:{l}_{3}}$ in the SWIR region which consists of $\:{l}_{1}$ ,$\:\:{l}_{2}$, and $\:{l}_{3}$ bands respectively. Also, the division should satisfy

1 $$\:{l}_{1}+{l}_{2}+{l}_{3}=d.$$

The auto-encoder neural network is applied to three partitions separately, as introduced in^5^, is composed of two sequential components known as encoder and decoder. The encoder component takes a multidimensional vector $\:{H}_{visible}\in\:{\text{R}}^{\text{h}\times\:\text{w}\times\:{l}_{1}}$ as input and produces a corresponding low-dimensional representation $\:{{H}^{\sim}}_{visible}\in\:{\text{R}}^{\text{h}\times\:\text{w}\times\:{m}_{1}}$, where $\:{m}_{1}<{l}_{1}$ the encoder consists of at least two fully connected layers. The first layer contains several neurons defined by the neural network’s architecture, each connected to all components of the input vector. The last layer of the encoder has neurons equal to the desired dimensionality of the reduced space.

The decoder typically mirrors the encoder’s architecture. It has the same number of layers with the same number of neurons, although this is not a strict requirement. The input layer of the decoder takes the reduced representation from the encoder’s output and reconstructs the original multidimensional data. The training process focuses on minimizing errors. The number of hidden layers and neurons is defined by the neural network’s architecture parameters.

After the application of autoencoders in the NIR region the sub-cube of $\:{{H}^{\sim}}_{NIR}\in\:{\text{R}}^{\text{h}\times\:\text{w}\times\:{m}_{2}}$ will be generated. Similarly, the sub-cube of $\:{{H}^{\sim}}_{SWIR}\in\:{\text{R}}^{\text{h}\times\:\text{w}\times\:{m}_{3}}$ will be generated in the SWIR region. If $\:p$ denotes total required bands after dimensionality reduction, the division should satisfy

2 $$\:{m}_{1}+{m}_{2}+{m}_{3}=p.$$

The HSI data cube after the application of dimensionality reduction is denoted by $\:{H}_{DR}\in\:{\text{R}}^{\text{h}\times\:\text{w}\times\:\text{p}}$ and can be denoted as below.

3 $$\:{H}_{DR}\in\:{\text{R}}^{\text{h}\times\:\text{w}\times\:\text{p}}={{H}^{\sim}}_{visible}\in\:{\text{R}}^{\text{h}\times\:\text{w}\times\:{m}_{1}}\:\cup\:\:{{H}^{\sim}}_{NIR}\in\:{\text{R}}^{\text{h}\times\:\text{w}\times\:{m}_{2}}\cup\:\:{{H}^{\sim}}_{SWIR}\in\:{\text{R}}^{\text{h}\times\:\text{w}\times\:{m}_{3}}.$$

Generally, Hyperspectral Imaging (HSI) data is characterized by the inherent properties of its underlying surface. Albedo Recovery (AR) is an intrinsic surface property determined by the material composition of the Earth, and it remains consistent regardless of imaging conditions and illumination. In this study, the Autoencoder Reconstruction (AR) is implemented using spatial filtering, which plays a crucial role in capturing meaningful semantic spatial information. This recovery process is applied to selected p bands of the HSI data represented as$\:{H}_{DR}\in\:{\text{R}}^{\text{h}\times\:\text{w}\times\:\text{p}}.$ Let x_k_ and x_k−1_ be neighboring pixels in the spatial domain. The domain transform of the data cube is given by

4 $$\:\varDelta\:={\int\:}_{{x}_{k-1}}^{{x}_{k}}1+\frac{{\sigma\:}_{s}}{{\sigma\:}_{r}}*\left|{H}_{DR}^{1}\left(x\right)\right|\:dx.$$

The isometric transformation, just as shown in (11) is designed to preserve the geodesic distance between neighboring pixels. Where $\:{\text{H}}_{\text{D}\text{R}}^{1}\left(\text{x}\right)$is the derivative of the discrete input intensity with respect to x, The spatial parameter is the $\:{\sigma\:}_{s}$ and the range parameter is $\:{\sigma\:}_{r}$. Let $\:{{H}_{DRAR}\in\:\text{R}}^{\text{h}\times\:\text{w}\times\:\text{p}}$ denotes Hyperspectral data after completion of the spatial filtering using AR.

Then, a Convolutional Neural Network (CNN) is employed to perform the task of Hyperspectral Image (HSI) classification. The CNN architecture consists of three primary layers. The first of these layers executes the convolution process, as illustrated below.

5 $$\:{\text{C}\text{O}\text{N}}_{\text{i},\text{j}}={\upsigma\:}{(\left(F{\otimes}\text{{\rm\:T}}\text{F}\right)}_{\text{i},\text{j}}+b).$$

In this perspective, the symbol ${\otimes}$ epitomizes the convolution operator, where *F* represents the filter, and $\:\left(\text{i},\text{j}\right)$ denotes the specific spatial location. $\:\text{{\rm\:T}}\text{F}$ represents the input training vector derived from the tensor $\:{{H}_{DRAR}\in\:\text{R}}^{\text{h}\times\:\text{w}\times\:\text{p}}$ and $\:b$ signifies the bias term. The function σ (.) indicates the activation function. In this study, ReLU is engaged, as represented below.

6 $$\:{\upsigma\:}\left(x\right)=\text{m}\text{a}\text{x}(0,x).$$

The subsequent layer involves pooling, which processes the feature maps generated by the convolutional layer. This model utilizes a max pooling layer to reduce the spatial dimensions of the feature maps, producing$\:{\text{P}\text{L}}_{\text{i},\text{j}}$, as output. After alternating applications of convolution and pooling layers, the feature maps are transformed into a flattened vector format, denoted as FLV^24^.

The third set comprises of the Fully Connected Layers, tasked with extracting deep and abstract-level features.

7 $$\:\text{O}\text{u}\text{t}\text{V}=\:\sum\:{\upsigma\:}\left({\upomega\:}\text{i}\text{*}\text{F}\text{L}\text{V}+{b}^{\sim}\right).$$

In the present context, σ (.) represents the activation function. The soft-max function is engaged at the final layer, while the residual layers operate ReLU as an activation function.$\:\:{b}^{\sim}$ is bias, $\:\text{O}\text{u}\text{t}\text{V}$ denotes output vector and $\:{\upomega\:}\text{i}$ weight.

**Fig. 2:**
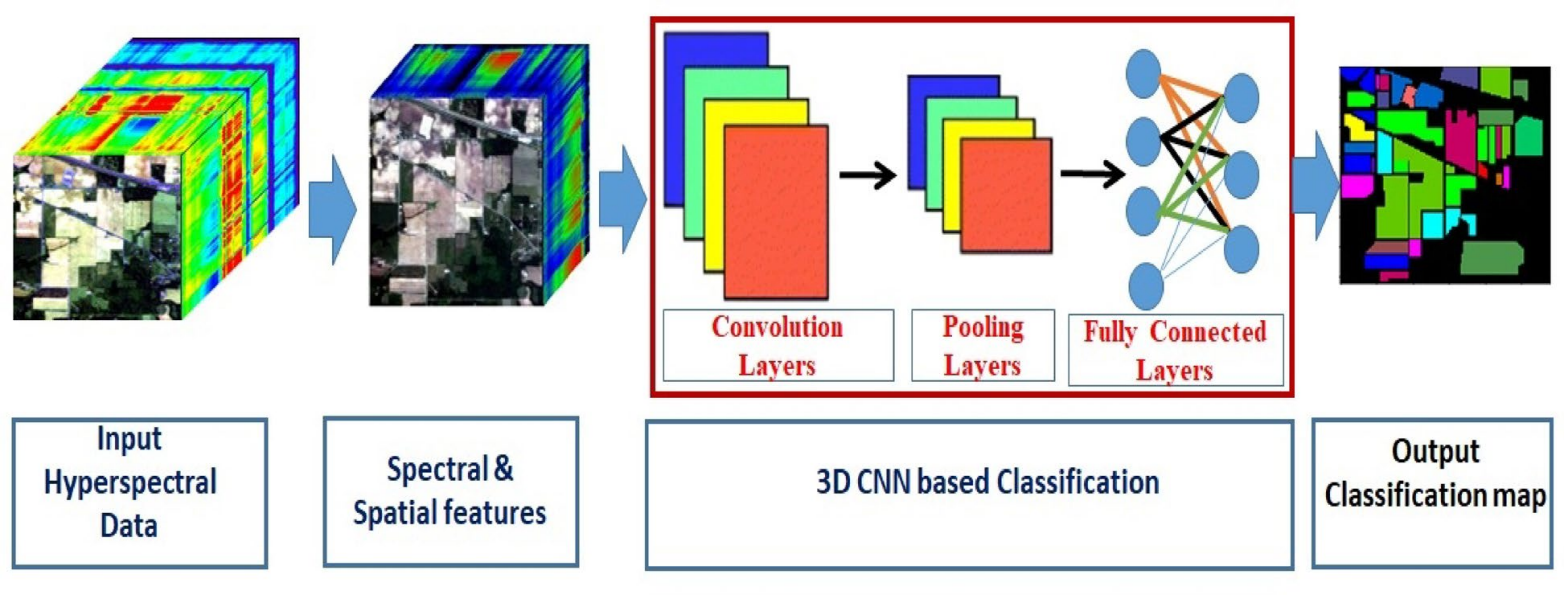
Methodology for hyperspectral image classification.

**Fig. 3:**
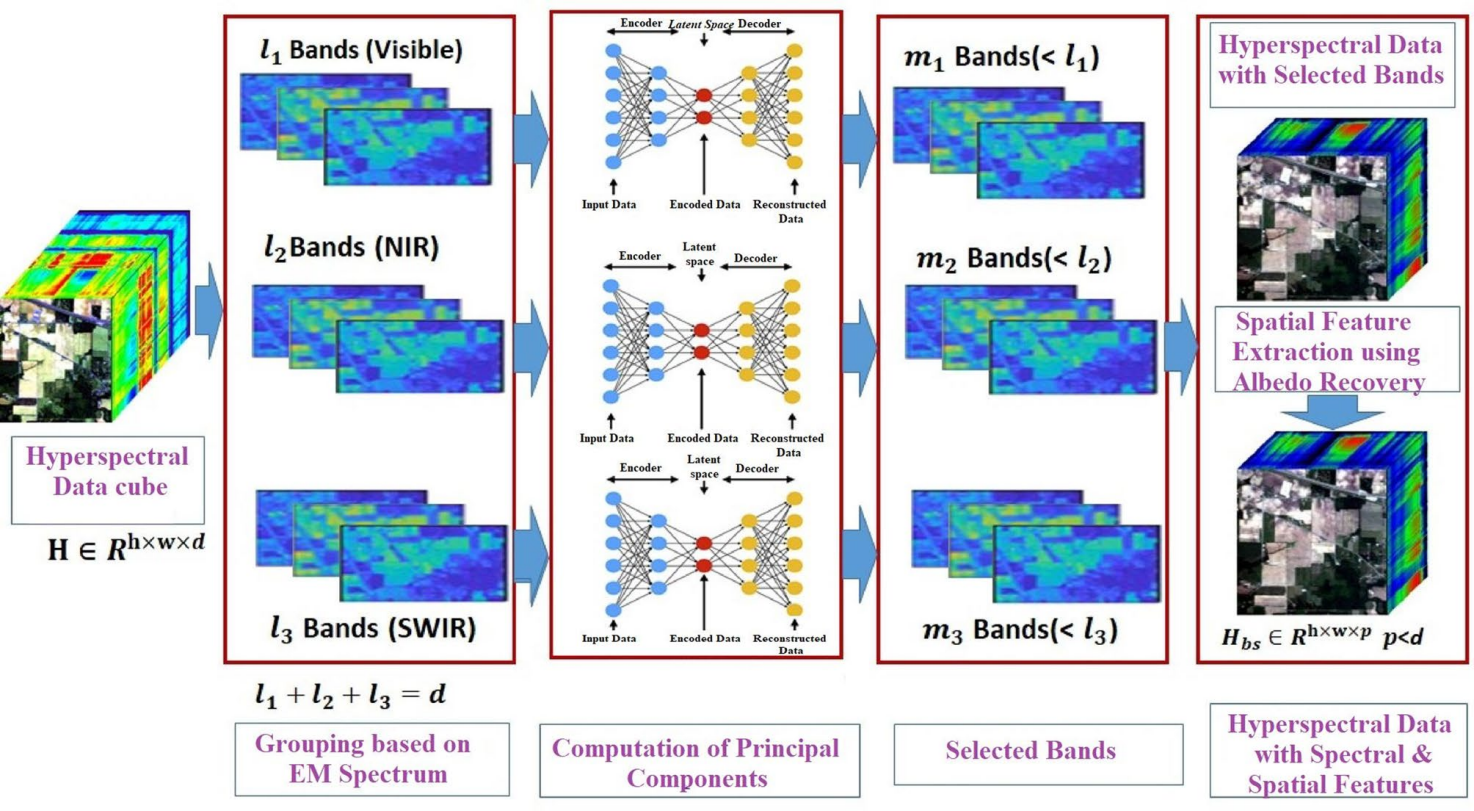
Band selection strategy for hyperspectral image classification.

The abstract model of the proposed methodology is shown in Fig. 2. A detailed description of various cascade blocks^25^ in the methodology is presented in Fig. 3. As illustrated in Fig. 3, the dimensionality reduction process begins with the spectral partitioning of the input hyperspectral image cube $\:\text{H}\in\:{\text{R}}^{\text{h}\times\:\text{w}\times\:\text{d}}$ into three physically meaningful spectral regions: VISIBLE, NIR, and SWIR, resulting in sub-cubes$\:{\text{H}}_{\text{visible}},{\text{H}}_{\text{NIR}}$ and $\:{\text{H}}_{\text{SWIR}}$ with band counts $\:{\text{l}}_{1},{\text{l}}_{2}\text{}$ and $\:\:{l}_{3}$ such that$\:{\:l}_{1}+{l}_{2}+\:{l}_{3}=d$

For each partition, an **independent autoencoder** is trained to learn a compact representation. The number of output bands $\:{m}_{1},\:{m}_{2}$, and $\:{m}_{3}$​ from each encoder is selected such that $\:{\text{m}}_{1}+{\text{m}}_{2}+{\text{m}}_{3}=\text{p}$, where p≪d

d represents the total number of bands in the final reduced representation

8 $$\:{H}_{DR}\in\:{R}^{h\times\:w\times\:p}.$$

**Band Allocation Strategy**.

The values of $\:{m}_{1},\:{m}_{2}$, and $\:{m}_{3}$​ were **not arbitrarily chosen** but determined through **empirical evaluation** based on classification performance (overall accuracy, average accuracy, and kappa coefficient) using a validation set for each dataset. The allocation strategy was as follows:

1. *Initial band proportioning* The number of bands from each spectral region was initially set **in proportion to the total number of bands** in that region (i.e., based on$\:{l}_{1},{l}_{2}\text{}$ and $\:\:{l}_{3}$).
2. *Fine-tuning through cross-validation* A grid search was conducted to slightly vary the values around this proportional baseline, ensuring the total sum remained p. The configuration yielding the **highest validation accuracy** was selected.
3. Final configurations
  **Indian Pines**: d = 200, *p* = 30, final configuration ($\:{m}_{1},\:{m}_{2},\:{m}_{3})=(\text{8,12,10})$
  **Salinas**: d = 204, *p* = 35, final configuration $\:{(m}_{1},\:{m}_{2},\:{m}_{3})=(\text{10,13,12})$
  **Pavia University**: d = 103, *p* = 20, final configuration $\:{(m}_{1},\:{m}_{2},\:{m}_{3})=(\text{6,7},7)$

These selections balance **spectral diversity preservation** and **dimensionality compactness**, with experimental results demonstrating improved classification performance.

### Validation strategy for band allocation.

To determine the optimal number of reduced bands $\:({m}_{1},\:{m}_{2},\:{m}_{3})$ from each spectral partition (VISIBLE, NIR, SWIR), we used a **cross-validation-based search strategy** that ensures the final dimensionality $\:{m}_{1}+{m}_{2}+{m}_{3}=p$ provides the best classification performance. The tuning process is formally outlined below.

The complete process of dimensionality reduction is discussed in Pseudocode 1.

**Pseudocode 1:**
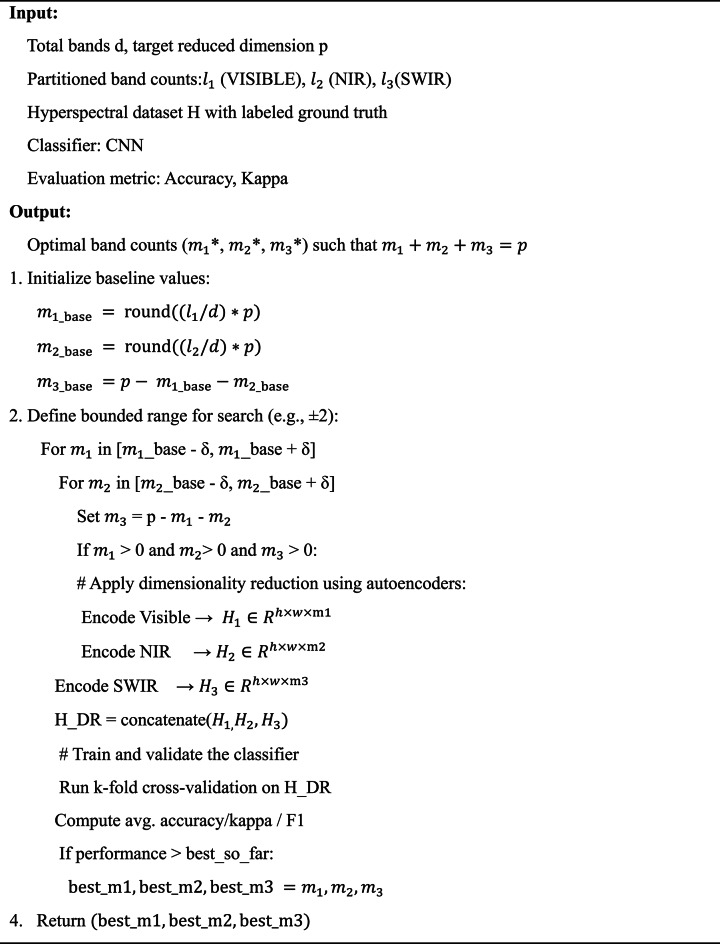
Optimal band allocation via cross-validation

- The tuning process is **dataset-specific** and repeated independently for Indian Pines, Salinas, and Pavia University.
- Classification performance is evaluated using **5-fold cross-validation** to ensure generalization.
- The best-performing configuration is selected for final dimensionality reduction and model training.

### Validation method for tuning $\:{m}_{1},\:{m}_{2},\:{m}_{3}$

To determine the optimal number of reduced bands from each spectral region—VISIBLE, NIR, and SWIR—we employed a **grid-based validation strategy** guided by classification performance. The goal was to identify the best combination of $\:{m}_{1},\:{m}_{2},\:{m}_{3}$​ that satisfies $\:{m}_{1}+{m}_{2}+{m}_{3}=p$

where $\:\text{p}$ is the total number of bands to retain after dimensionality reduction.

The validation procedure consists of the following steps:

#### Initial band ratio estimation

We begin by estimating the initial values of $\:{m}_{1},\:{m}_{2},\:{m}_{3}\:$Based on the proportion of spectral bands in each region:

9 $$\:{m}_{i}=\left\lfloor \frac{{l}_{i}}{d}\cdot\:p \right\rfloor,\hspace{1em}\text{for\:}i\in\:\{\text{1,2},3\}.$$

where$\:{l}_{i}$is the number of original bands in region I and $\:\text{d}={\text{l}}_{1}+{\text{l}}_{2}+{\text{l}}_{3}$ ​. This proportional allocation ensures that each region contributes according to its spectral richness.

#### Search space construction

Next, we construct a search space around the initial estimate by varying each $\:{m}_{\text{i}}$ within a small bounded range (e.g., ± 2 or ± 3), ensuring that the sum $\:{m}_{1}+{m}_{2}+{m}_{3}=p$ is always maintained.

#### Cross-validation framework

For each valid combination of $\:({m}_{1},\:{m}_{2},\:{m}_{3})$, the following steps are repeated:

- Apply autoencoder-based dimensionality reduction using the candidate $\:{m}_{\text{i}}\:$values.
- Train a lightweight classifier (e.g., SVM, Random Forest, or CNN) using the resulting reduced HSI cube.
- Evaluate classification performance using **k-fold cross-validation**(typically k = 5) on the labeled ground truth data.

The performance metrics considered include:

- Overall Accuracy (OA).
- Average Accuracy (AA).
- Kappa Coefficient.
- F1-Score.

#### Optimal selection criteria

The combination $\:\left({\text{m}}_{1}^{\text{*}},{\text{m}}_{2}^{\text{*}},{\text{m}}_{3}^{\text{*}}\right)\:\:\:\:$that yields the **highest cross-validation performance**(primarily OA and Kappa) is selected as the final configuration for that dataset.

## Description of hyperspectral datasets

**Fig. 4:**
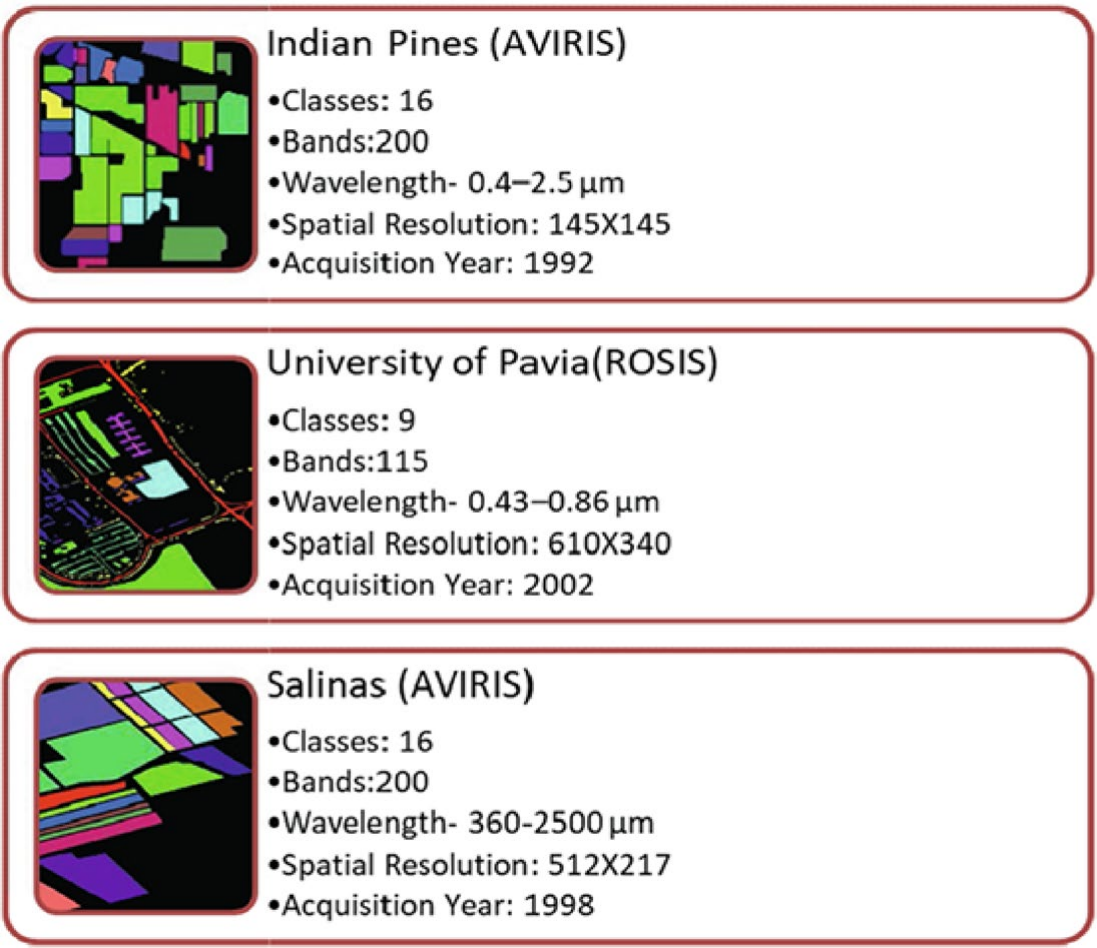
Glimpse of Hyperspectral Datasets for Image Classification.

In the present work, to perform all the experiments and evaluate the models, three real public hyperspectral image datasets are used. There are a lot of variances among the datasets in terms of spectral, spatial, and other properties. The complete description of the three datasets^26,44–48,52,54^ is compiled as shown in Fig. 4.

## Parameter tuning

This section is to describe the parameter tuning in the different phases of the proposed method. Indian Pines and Salinas datasets are acquired from the AVIRIS sensor. Thus, the Visible 400–700 nm region bands are Band 1–33, Near-infrared (NIR) 700–1000 nm region bands are Band 34–64 and Short-wave infrared (SWIR) 1000–2500 nm region bands are Band 65–200. University of Pavia data set is acquired from ROSIS sensor. Thus, the Visible 400–700 nm region bands are Band 1–64, and Near-infrared (NIR) 700–1000 nm region bands are Band 65–103. Based on this information, $\:{l}_{1},{l}_{2,}{l}_{3}$ values are considered^27^.

**Table 1:** Parameters used in dimensionality reduction.

Name of the dataset	$\:{l}_{1}$	$\:{l}_{2}$	$\:{l}_{3}$	$\:{m}_{1}$	$\:{m}_{2}$	$\:{m}_{3}$	$\:p$
Indian Pines	33	31	136	11	15	24	14
University of Pavia	64	39	0	9	25	0	12
Salinas	33	31	136	12	13	21	16

The ROSIS (Reflective Optics System Imaging Spectrometer) sensor, which only detects in the visible to near-infrared (VNIR) range, roughly between 0.43 and 0.86 μm, and does not detect in the SWIR region (usually > 1.0 μm), was used to collect the PU dataset. Because of this, the initial hyperspectral image does not contain any SWIR bands, making them unavailable for selection or analysis (Table 1).

The proposed classification network is designed as a compact CNN architecture optimized for hyperspectral image classification. The architecture consists of 3 convolutional layers, each followed by batch normalization, ReLU activation, dropout, and max pooling. These are followed by a fully connected (dense) network of 8 layers (1 input, 6 hidden, and 1 output layer). The full model configuration is detailed in Table 2, and the training parameters configuration is given in Table 3.

**Table 2:** Detailed CNN architecture and layer-wise settings.

Layer No.	Layer type	Parameters
1	Input Layer	Input size: h×w×p (where p is reduced bands)
2	Convolutional Layer	Filters: 32, Kernel: 3 × 3, Stride: 1, Padding: ‘Same’, Activation: ReLU
3	Batch Normalization	-
4	Max Pooling	Pool Size: 2 × 2, Stride: 2
5	Dropout	Rate: 0.3
6	Convolutional Layer	Filters: 64, Kernel: 3 × 3, Stride: 1, Padding: ‘Same’, Activation: ReLU
7	Batch Normalization	-
8	Max Pooling	Pool Size: 2 × 2, Stride: 2
9	Dropout	Rate: 0.3
10	Convolutional Layer	Filters: 128, Kernel: 3 × 3, Stride: 1, Padding: ‘Same’, Activation: ReLU
11	Batch Normalization	-
12	Max Pooling	Pool Size: 2 × 2, Stride: 2
13	Dropout	Rate: 0.3
14	Flatten	Converts 3D feature map to 1D
15	Dense Layer	Neurons: 512, Activation: ReLU
16	Dense Layer	Neurons: 256, Activation: ReLU
17	Dense Layer	Neurons: 128, Activation: ReLU
18	Dense Layer	Neurons: 64, Activation: ReLU
19	Dense Layer	Neurons: 32, Activation: ReLU
20	Dense Layer	Neurons: 16, Activation: ReLU
21	Output Layer	Neurons: CC (number of classes), Activation: Softmax

**Table 3:** Training parameters configuration.

Parameter	Value
Batch Size	50
Epochs	100
Optimizer	Adam
Learning Rate	0.001
Loss Function	Categorical Cross-Entropy
Dropout	Enabled
Batch Normalization	Enabled

The number of selected spectral bands, p, and the spatial patch sizes ​$\:{m}_{1},\:{m}_{2},\:{m}_{3},$ play a crucial role in balancing spectral-spatial information and reducing computational complexity. In this study, these parameters were **empirically determined** through a systematic tuning procedure.

For each dataset—**Indian Pines**, **University of Pavia**, and **Salinas**—a range of candidate values for p (e.g., 8 to 24 bands) was evaluated. For each value, a subset of bands was selected using our band selection module, and classification accuracy was assessed using a fixed validation split. The value of p that yielded the highest validation performance was chosen for final use: 14 bands for Indian Pines, 12 bands for University of Pavia, and 16 bands for Salinas.

Similarly, spatial window sizes (m×m) were varied (e.g., 9 × 9, 11 × 11, 13 × 13, etc.) and tested alongside the selected spectral bands. The evaluation was conducted using **5-fold cross-validation**, ensuring robustness and generalization. The spatial window sizes were selected based on the configuration that maximized average classification performance across folds, resulting in dataset-specific values for ​$\:{m}_{1},\:{m}_{2},\:{m}_{3}.$

These empirically optimized configurations ensure a good trade-off between preserving meaningful spectral-spatial features and computational efficiency. Table 1 summarizes the final values of p and m for each dataset.

## Results and observations

**Fig. 5:**
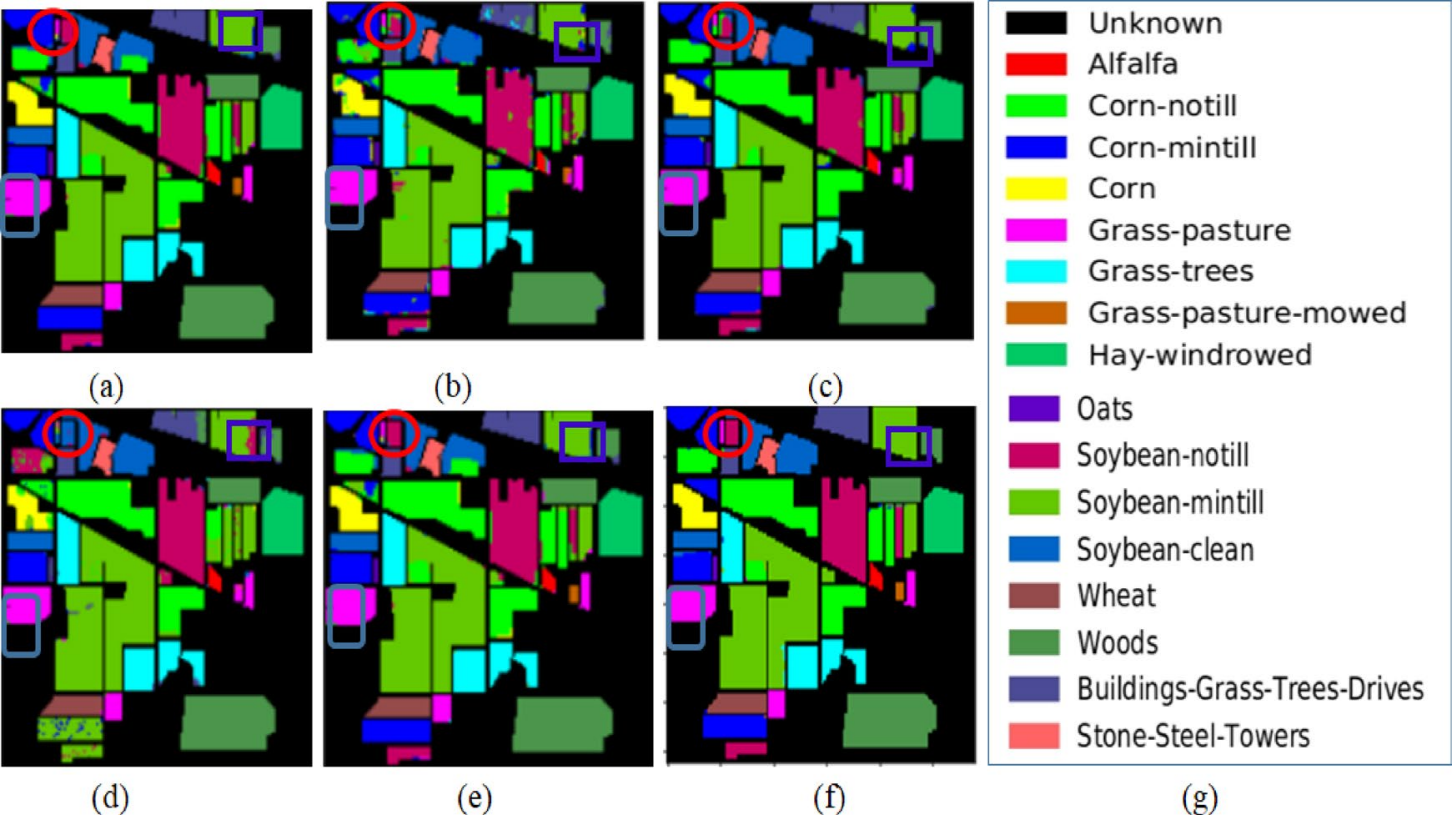
Results of the classification maps obtained from the Indian Pines Hyperspectral data (**a**) Ground map (**b**) FNPCW (**c**) AEC (**d**) UBS (**e**) UBS-CL (**f**) proposed method (**g**) category label.

The proposed approach’s effectiveness is validated by contrasting it with the latest methods in the field. For a fair comparison, we have chosen the state-of-the-art methods which are closer in the methodology as compared with the proposed method. The initial comparison method employs Hyperspectral image classification using a FNPCW approach^29^. This method integrates fuzzy-embedded hyperbolic sigmoid nonlinear principal component analysis with a weighted least squares technique. Here, principal component analysis is treated as a fuzzy optimization problem to extract spectral band information effectively. Spatial filtering is then applied to each extracted principal component to reduce inherent noise. Finally, spectral and spatial features are fused and trained using support vector machine algorithms, with Gaussian kernel applied to generate classification maps. Notably, this method achieves high accuracy as compared with state-of-the-art methods. The qualitative results are shown in Fig. 5 (Grass-pasture, Soybean-notill and Soybean-mintill are highlighted), and the quantitative results are in Table 4.

**Fig. 6:**
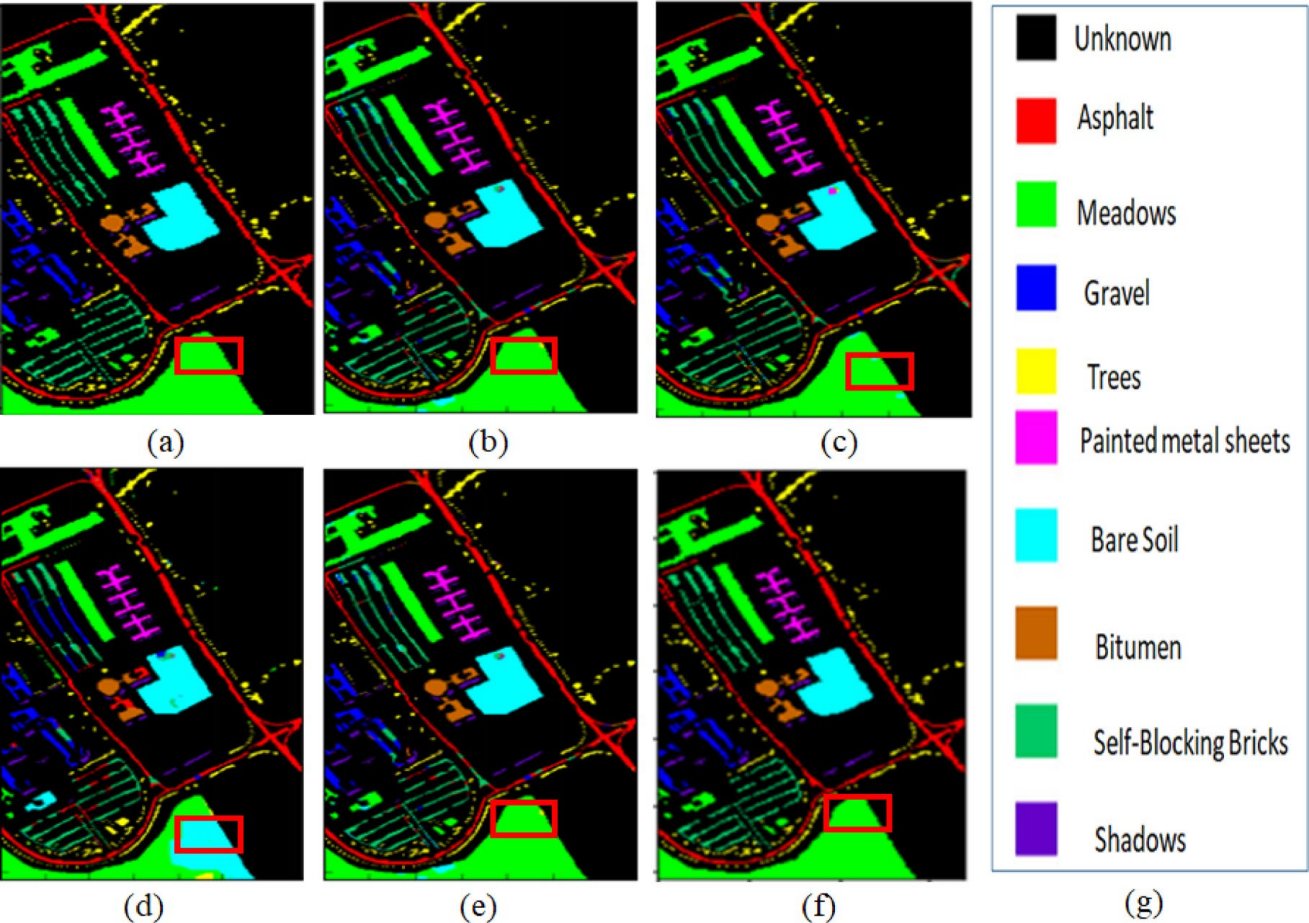
Results of the classification maps obtained from the University of Pavia Hyperspectral data (**a**) Ground map (**b**) FNPCW (**c**) AEC (**d**) UBS (**e**) UBS-CL (**f**) proposed method (**g**) category label.

The second comparative method is based on autoencoders, and clustering called AEC^30^. This method introduces a novel unsupervised band selection method leveraging deep learning frameworks to address the challenges of hyperspectral imaging classification. The proposed approach consists of two phases: unmixing and clustering. In the unmixing phase, a nonlinear deep autoencoder accurately extracts material spectra, while in the clustering phase; variance vectors are generated and clustered using classical K-means. The qualitative results are shown in Fig. 6 (Meadows class is highlighted), and the quantitative results are in Table 5.

**Fig. 7:**
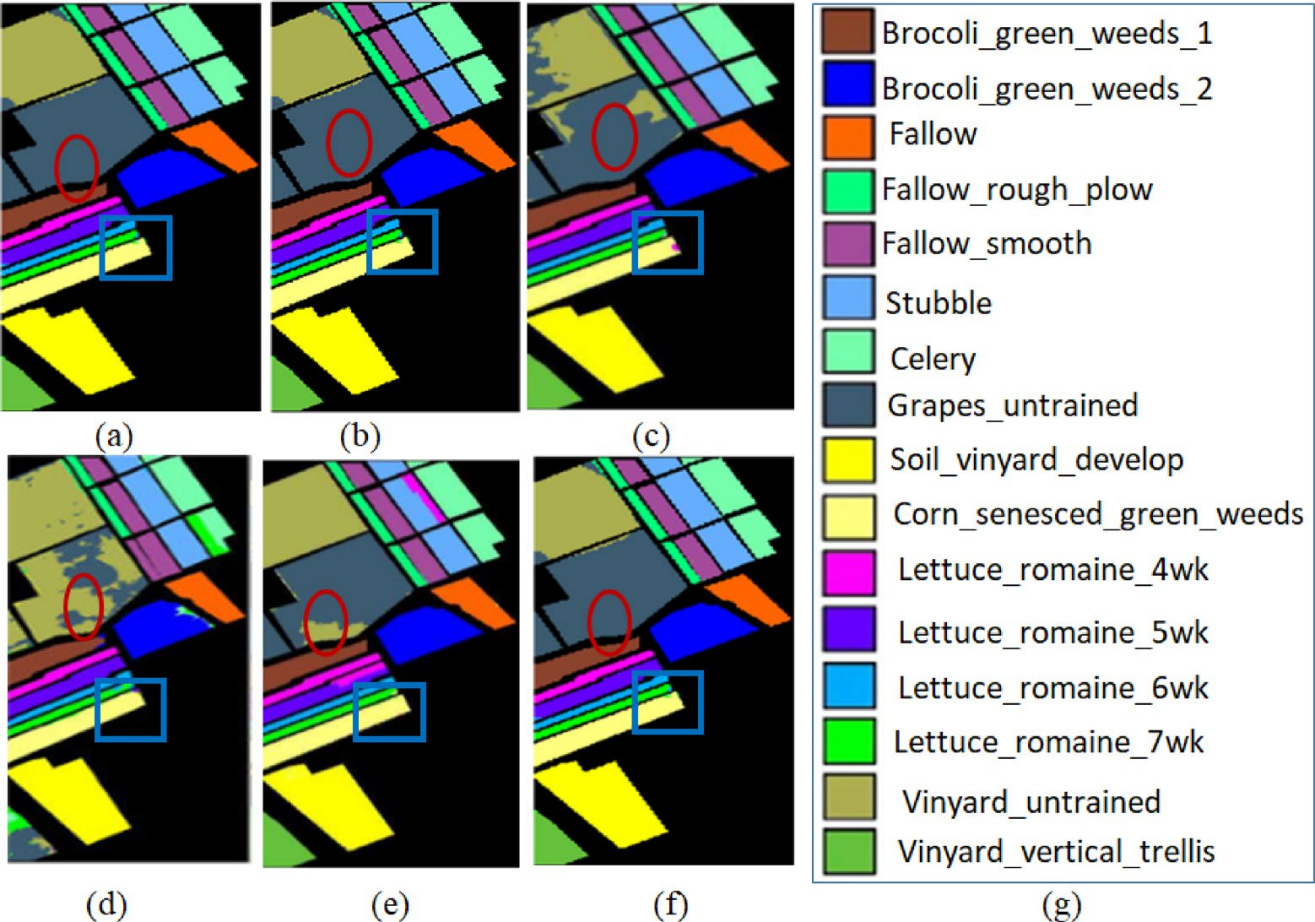
Results of the classification maps obtained from the Salinas Hyperspectral data (**a**) Ground map (**b**) FNPCW (**c**) AEC (**d**) UBS (**e**) UBS-CL (**f**) proposed method (**g**) category label.

The third comparative method is for improving hyperspectral image (HSI) classification accuracy by integrating spectral and spatial features. This approach (UBS)^31^ is based on unsupervised band selection to extract a subset of spectral bands while preserving data structure. Spatial features are extracted using a Structure-Preserving Recursive Filter (SPRF). Convolutional Neural Networks is employed with various layers for classification. Experimental results on Indian Pines, University of Pavia, and Salinas datasets demonstrate superior performance in terms of Overall Accuracy (OA), Average Accuracy (AA), and kappa coefficient (k)^32^. The qualitative results^33^ are shown in figure-7 (Grapes_untrained and Corn_senesced_green_weeds classes are highlighted), and the quantitative results^34^ are in Table 6.

**Table 4:** Comparative classification results on Indian Pines Dataset.

Method	OA (%) ± SD	AA (%) ± SD	Kappa ± SD
FNPCW^29^	89.12 ± 0.87	88.54 ± 0.90	87.23 ± 0.89
AEC^30^	91.34 ± 0.74	90.29 ± 0.85	89.11 ± 0.84
UBS^31^	90.76 ± 0.80	89.98 ± 0.88	88.76 ± 0.86
UBS-CL^35^	92.11 ± 0.71	91.45 ± 0.80	90.02 ± 0.78
PFS3F^17^	93.45 ± 0.65	92.60 ± 0.72	91.44 ± 0.70
Transformer + SimAM^18^	94.02 ± 0.60	93.12 ± 0.68	91.92 ± 0.66
DB-SFE^19^	94.66 ± 0.55	93.85 ± 0.60	92.58 ± 0.58
**Proposed Method**	**95.34 ± 0.50**	**94.50 ± 0.55**	**93.20 ± 0.52**

**Table 5:** Comparative classification results on the Salinas Dataset.

Method	OA (%) ± SD	AA (%) ± SD	Kappa ± SD
FNPCW^29^	91.23 ± 0.85	90.88 ± 0.86	90.00 ± 0.84
AEC^30^	92.78 ± 0.72	92.00 ± 0.78	91.75 ± 0.70
UBS^31^	92.30 ± 0.75	91.74 ± 0.79	91.30 ± 0.72
UBS-CL^35^	93.55 ± 0.66	93.00 ± 0.69	92.80 ± 0.65
PFS3F^17^	94.40 ± 0.58	94.00 ± 0.60	93.70 ± 0.57
Transformer + SimAM^18^	94.95 ± 0.54	94.60 ± 0.56	94.30 ± 0.52
DB-SFE^19^	95.34 ± 0.50	95.05 ± 0.52	94.90 ± 0.50
**Proposed Method**	**96.10 ± 0.47**	**95.80 ± 0.48**	**95.60 ± 0.46**

**Table 6:** Comparative classification results on Pavia university Dataset.

Method	OA (%) ± SD	AA (%) ± SD	Kappa ± SD
FNPCW^29^	90.45 ± 0.88	89.50 ± 0.87	88.90 ± 0.86
AEC^30^	91.92 ± 0.76	90.78 ± 0.79	90.40 ± 0.75
UBS^31^	91.60 ± 0.78	90.30 ± 0.81	90.00 ± 0.76
UBS-CL^35^	92.88 ± 0.69	91.85 ± 0.71	91.50 ± 0.68
PFS3F^17^	93.70 ± 0.61	92.65 ± 0.62	92.30 ± 0.60
Transformer + SimAM^18^	94.26 ± 0.56	93.15 ± 0.58	92.90 ± 0.55
DB-SFE^19^	94.85 ± 0.53	93.75 ± 0.54	93.55 ± 0.52
**Proposed Method**	**95.55 ± 0.49**	**94.45 ± 0.50**	**94.25 ± 0.48**

Method via Contrastive Learning for Hyperspectral Images called UBS-CL^35^. This method uses contrastive learning-based unsupervised band selection to elevate nonlinear relationships on Hyperspectral data. This method got an overall accuracy of 97.2%, 94.3%, and 96% for Indian Pines, University of Pavia, and Salinas datasets, respectively (Fig. 7).

To show the effectiveness of the proposed method ablation test is performed. The results of the ablation test are presented in Table 7. The major modules in the present work include Initial Grouping, Non-Linear Feature Extraction, and Albedo Recovery. An ablation study is conducted for all three datasets by varying different combinations of the modules^36^. The highest OA values of 98.19%, 97.92%, and 99.93% are reported by the inclusion of all the modules for the Indian Pines, University of Pavia, and Salinas datasets, respectively. If Albedo recovery is excluded from the experiment, the results are closer to the highest OA values. The least OA values of 82.32%, 81.33%, and 78.7% are reported by the exclusion of all the modules except the Albedo recovery module for the Indian Pines and Salinas datasets, respectively. This clearly shows the importance of Initial Grouping and Non-Linear Feature Extraction in band selection^37^. The moderate results are reported by the inclusion of either Initial Grouping or Non-Linear Feature Extraction. Modules like Initial Grouping and Non-Linear Feature Extraction^38^ are super helpful in selecting appropriate bands and thus achieving the highest classification accuracy^39^.

**Table 7:** Results of the ablation test.

Initial Grouping	Non-Linear Feature Extraction	Albedo recovery	Overall accuracy (OA)	Average accuracy (AA)	Kappa coefficient (κ)
**Indian Pines**					
✓	✓	✓	98.19	97.24	96.99
✓	✓		97.02	96.23	97.04
✓		✓	98	96.12	95
	✓	✓	97.14	96.9	94
		✓	82.32	85	84
**University of Pavia**					
✓	✓	✓	97.92	97	96
✓	✓		97.14	94	93
✓		✓	96.87	96	94
	✓	✓	95.83	94.88	93.49
		✓	81.33	80.44	81
**Salinas**					
✓	✓	✓	99.93	99.1	98.45
✓	✓		98.14	98.33	97.88
✓		✓	97.87	97.04	95.99
	✓	✓	94.83	93.05	92.9
		✓	78.7	78	76.44

The software used in this work includes MATLAB R2020b and Python with Jupyter Notebook. The hardware consists of a PC equipped with an Intel(R) Core (TM) i5-6500 CPU running at 3.20 GHz and 16 GB of RAM.

**Fig. 8:**
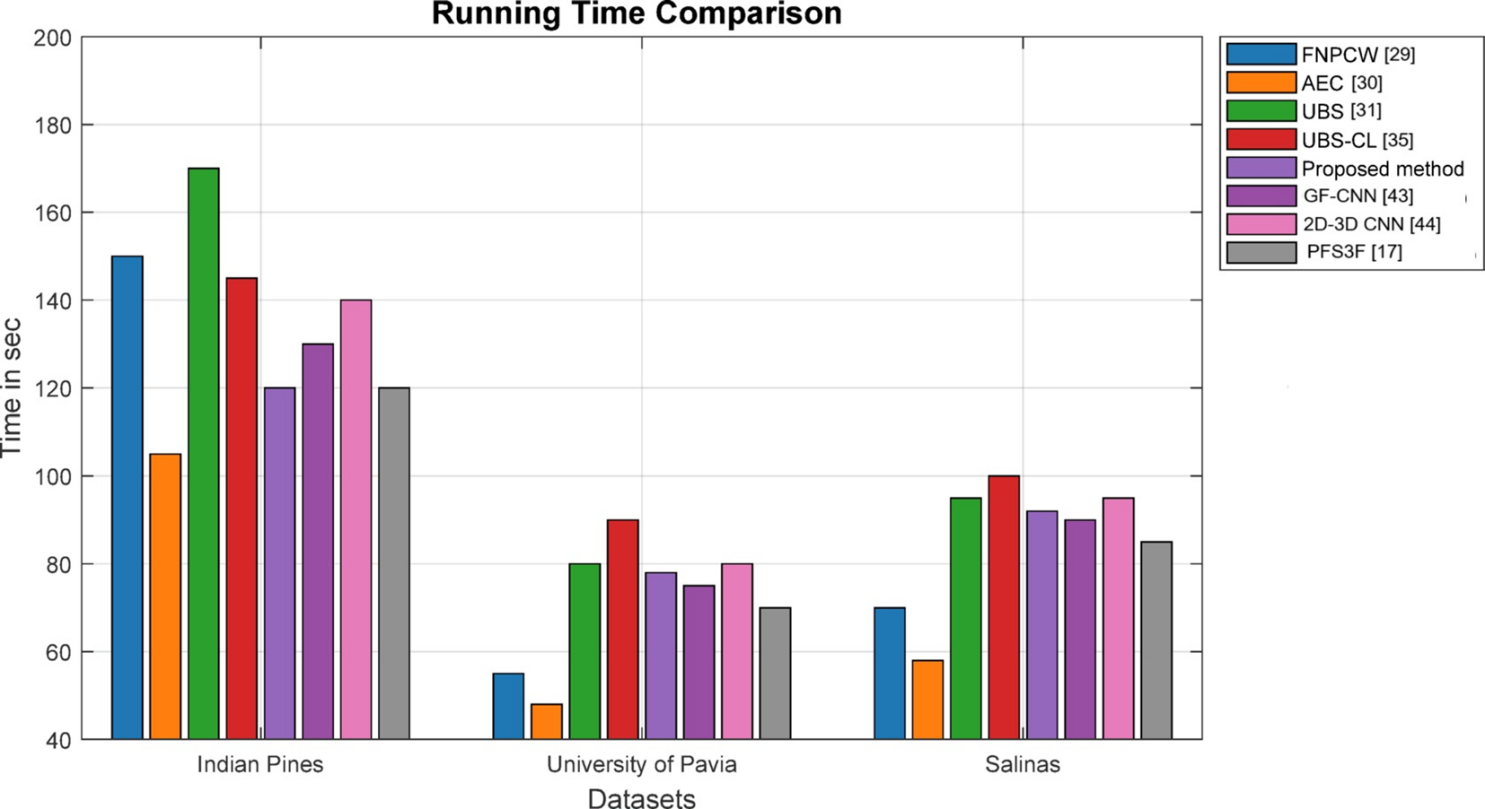
Comparison of running time complexity.

To further validate the efficacy of the proposed method, the time complexity is calculated for each of the methods across four datasets. Figure 8 shows the comparison of the proposed method concerning running time^40^ against the state-of-the-art methods. AEC consistently shows the lowest running time, especially on University of Pavia and Salinas datasets, due to its efficient shallow architecture. The proposed Transformer-based model achieves a good balance, being faster than UBS^31^/UBS-CL^35^ and close to Zhang et al. (2025)^17^ in efficiency.

## Conclusions and future work

In conclusion, our study presents a comprehensive framework for hyperspectral image processing, addressing key challenges such as high dimensionality, spectral-spatial resolution trade-offs, and classification accuracy.  By incorporating innovative techniques such as electromagnetic spectrum-based grouping and autoencoder-driven non-linear feature extraction, this work achieves notable improvements in both dimensionality reduction and albedo recovery ^41^. Comprehensive experiments conducted on a range of hyperspectral datasets—namely Indian Pines, University of Pavia, and Salinas—demonstrate the effectiveness and adaptability of the proposed method. The results indicate superior performance in both dimensionality reduction and spatial enhancement compared to existing methods, thereby providing a solid foundation for more accurate interpretation and analysis of hyperspectral imagery^42^. By effectively integrating the complementary strengths of spectral and spatial features, the proposed method provides a comprehensive approach for extracting valuable insights from hyperspectral imagery. Looking forward, future research may focus on exploring more advanced deep learning architectures, incorporating richer contextual information, and expanding the framework’s applicability to diverse remote sensing modalities^43^. Ultimately, our work contributes to the ongoing advancement of hyperspectral image processing, paving the way for enhanced understanding and utilization of this valuable technology in diverse scientific and practical domains.

## Data Availability

The datasets generated and/or analyzed during the current study are available in the Hyperspectral Remote Sensing Scenes repository “Hyperspectral Remote Sensing Scenes - Grupo de Inteligencia Computacional (GIC)”https://www.ehu.eus/ccwintco/index.php/Hyperspectral_Remote_Sensing_Scenes.
