# Correction: A new band selection approach integrated with physical reflectance autoencoders and albedo recovery for hyperspectral image classification

**DOI:** 10.1038/s41598-025-31906-1

**Published:** 2025-12-11

**Authors:** V. Sangeetha, L. Agilandeeswari

**Affiliations:** School of Computer Science Engineering & Information Systems, Vellore Institute of Technology, Vellore, 632014 India

Correction to: *Scientific Reports* 10.1038/s41598-025-09355-7, published online 31 July 2025

In the original version of this article, a description of Figures 5 and 7 was missing under the Results and observations section.

As a result, where:

“The qualitative results are shown in Fig. 5 (Corn and Soybean mintill classes are highlighted) and the quantitative results are in Table 4.”

now reads:

“The qualitative results are shown in Fig. 5 (Grass-pasture, Soybean-notill and Soybean-mintill are highlighted), and the quantitative results are in Table 4.”

And where:

“The qualitative results^33^ are shown in Figure 7 (Lettuce_romaine_4wk class is highlighted) and the quantitative results^34^ are in Table 6.”

now reads,

The qualitative results^33^ are shown in Figure 7 (Grapes_untrained and Corn_senesced_green_weeds classes are highlighted), and the quantitative results^34^ are in Table 6.

The original Article has been corrected.

